# INTERGENERATIONAL TRANSMISSION OF "SEVA": ELDERS MODEL SELFLESS SERVICE IN SOUTH ASIAN HOMES FOR CHILDREN

**DOI:** 10.1093/geroni/igac059.2140

**Published:** 2022-12-20

**Authors:** Connie Corley, Laura Sherwood, Dibendu Ghosh, David Willis

**Affiliations:** Fielding Graduate University, Santa Barbara, California, United States; Fielding Graduate University, Santa Barbara, California, United States; Fielding Graduate University, Santa Barbara, California, United States; Fielding Graduate University, Santa Barbara, California, United States

## Abstract

Life course theory is a framework for examining the role of elders in modeling altruistic behavior or "seva" (selfless service) in two homes for children: Unatti (Bhaktapur, Nepal) and Ramana's Garden (Rishikesh, India). Two American women, compelled to impact the plight of children subject to poverty, trafficking and/or caste discrimination (Dalits, the former "untouchables"), each founded homes 20+ years ago and modeled selfless service exemplified by children, some now in "emerging adulthood," who are giving back to their communities. In this intergenerational/intercultural multiple case study, narratives of the program founders now in mid- to late adulthood are presented along with narratives of six young adults who continue to provide nurturance to younger children and engage in projects to educate and feed residents of the homes and nearby communities. The life course principle of time and place situates the children in areas of their countries with relatively low literacy rates. The founders saw the value of education to empower young people, especially girls, to expand their opportunities and serve as role models for children coming into the homes after them. The linked lives principle is evident through shared relationships with the founders as these young people navigate political crises, disasters like the Nepal earthquake, and health crises like the Covid-19 pandemic. Some become leaders themselves as they have been mentored over their life course. A sense of agency is experienced in their young adulthood as more choices for their lives become available as an alternative to subsistence existence and early marriage/childbearing.

